# *Chlamydia trachomatis* fails to protect its growth niche against pro-apoptotic insults

**DOI:** 10.1038/s41418-018-0224-2

**Published:** 2018-10-30

**Authors:** Barbara S. Sixt, Carlos Núñez-Otero, Oliver Kepp, Raphael H. Valdivia, Guido Kroemer

**Affiliations:** 1 Laboratory for Molecular Infection Medicine Sweden, Umeå Centre for Microbial Research, Department of Molecular Biology, Umeå University, 90187 Umeå, Sweden; 2 INSERM U1138, Centre de Recherche des Cordeliers, 75006 Paris, France; 3 Equipe 11 labellisée Ligue Nationale contre le Cancer, Centre de Recherche des Cordeliers, 75006 Paris, France; 4 Université Paris Descartes, 75006 Paris, France; 5 Metabolomics and Cell Biology Platforms, Institut Gustave Roussy, 94800 Villejuif, France; 6 Laboratory for Molecular Infection Medicine Sweden, Umeå Centre for Microbial Research, Department of Clinical Microbiology, Umeå University, 90185 Umeå, Sweden; 7 Department of Molecular Genetics and Microbiology, Duke University School of Medicine, Durham, NC 27710 USA; 8 Pôle de Biologie, Hôpital Européen Georges-Pompidou, AP-HP, 75015 Paris, France; 9 Department of Women’s and Children’s Health, Karolinska Institute, Karolinska University Hospital, 17176 Stockholm, Sweden

**Keywords:** Infectious diseases, Cell death and immune response

## Abstract

*Chlamydia trachomatis* is an obligate intracellular bacterial agent responsible for ocular infections and sexually transmitted diseases. It has been postulated that *Chlamydia* inhibits apoptosis in host cells to maintain an intact replicative niche until sufficient infectious progeny can be generated. Here we report that, while cells infected with *C. trachomatis* are protected from apoptosis at early and mid-stages of infection, they remain susceptible to the induction of other cell death modalities. By monitoring the fate of infected cells by time-lapse video microscopy and by analyzing host plasma membrane integrity and the activity of caspases, we determined that *C. trachomatis*-infected cells exposed to pro-apoptotic stimuli predominately died by a mechanism resembling necrosis. This necrotic death of infected cells occurred with kinetics similar to the induction of apoptosis in uninfected cells, indicating that *C. trachomatis* fails to considerably prolong the lifespan of its host cell when exposed to pro-apoptotic insults. Inhibitors of bacterial protein synthesis partially blocked necrotic death of infected cells, suggesting that the switch from apoptosis to necrosis relies on an active contribution of the bacteria. Tumor necrosis factor alpha (TNF-α)-mediated induction of necrosis in cells infected with *C. trachomatis* was not dependent on canonical regulators of necroptosis, such as RIPK1, RIPK3, or MLKL, yet was blocked by inhibition or depletion of CASP8. These results suggest that alternative signaling pathways regulate necrotic death in the context of *C. trachomatis* infections. Finally, consistent with the inability of *C. trachomatis* to preserve host cell viability, necrosis resulting from pro-apoptotic conditions significantly impaired production of infectious progeny. Taken together, our findings suggest that *Chlamydia’s* anti-apoptotic activities are not sufficient to protect the pathogen’s replicative niche.

## Introduction

*C. trachomatis* is the causative agent of blinding trachoma, an ocular disease that is endemic in many developing countries [1]. Moreover, *C. trachomatis* is the most frequent agent of bacterial sexually transmitted disease worldwide [2]. Acute *Chlamydia* urogenital tract infections are often asymptomatic, but repeated and recurrent infections increase the risk for complications, such as pelvic inflammatory disease, ectopic pregnancy, and infertility [3].

*C. trachomatis’* replication is restricted to the intracellular environment of epithelial cells [4]. Within the host cell, *C. trachomatis* undergoes a developmental cycle, alternating between the reticulate body (RB) that replicates within an intracellular membrane-bound compartment termed inclusion and the elementary body (EB) that is eventually released from the host cell to infect neighboring cells [5]. Bacterial egress occurs via extrusion, which is a process that is non-destructive for the host cell, or via induction of a caspase-independent mode of host cell death that can be accompanied by necrotic and/or apoptotic morphological features [6–8].

At early and mid-stages of infection, cells infected with *Chlamydia* spp. are protected from the induction of apoptosis upon exposure to potent inducers [9], including for instance UV irradiation, cytotoxic chemicals (e.g., staurosporine (STS)), and immune mediators (e.g., tumor necrosis factor alpha (TNF-α) and ligation of CD95) [10, 11]. It has been proposed that the apoptotic machinery in *C. trachomatis*-infected cells is blocked upstream of the permeabilization of the mitochondrial outer membrane. Indeed, activation of BAX/BAK and the release of mitochondrial cytochrome *c* do not to occur in infected cells upon exposure to pro-apoptotic stimuli [10–14]. Infection with *C. trachomatis* also blocks the activation of apoptotic caspases, PARP cleavage, and pyknosis [10–13]. Accordingly, multiple anti-apoptotic activities have been attributed to *C. trachomatis*. These include for instance the stabilization of MCL-1 [15], downregulation and degradation of TP53 [16, 17], and enhanced recruitment of hexokinase-II to mitochondria [18]. Together these anti-apoptotic activities are predicted to protect the pathogen’s replicative niche from cytotoxic insults, such as from infection-induced stress and death signals emanating from immune cells.

Here we demonstrate that under pro-apoptotic conditions the death of *Chlamydia*-infected cells was not abolished, but rather shifted from apoptosis to an atypical form of necrosis. We further provide evidence that this necrotic death partially relies on an active contribution of the bacteria and that *C. trachomatis* fails to generate infectious progeny under pro-apoptotic conditions.

## Results

### Treatment with STS fails to activate apoptotic effector caspases in *C. trachomatis*-infected cells but still leads to host cell lysis

**Fig. 1:**
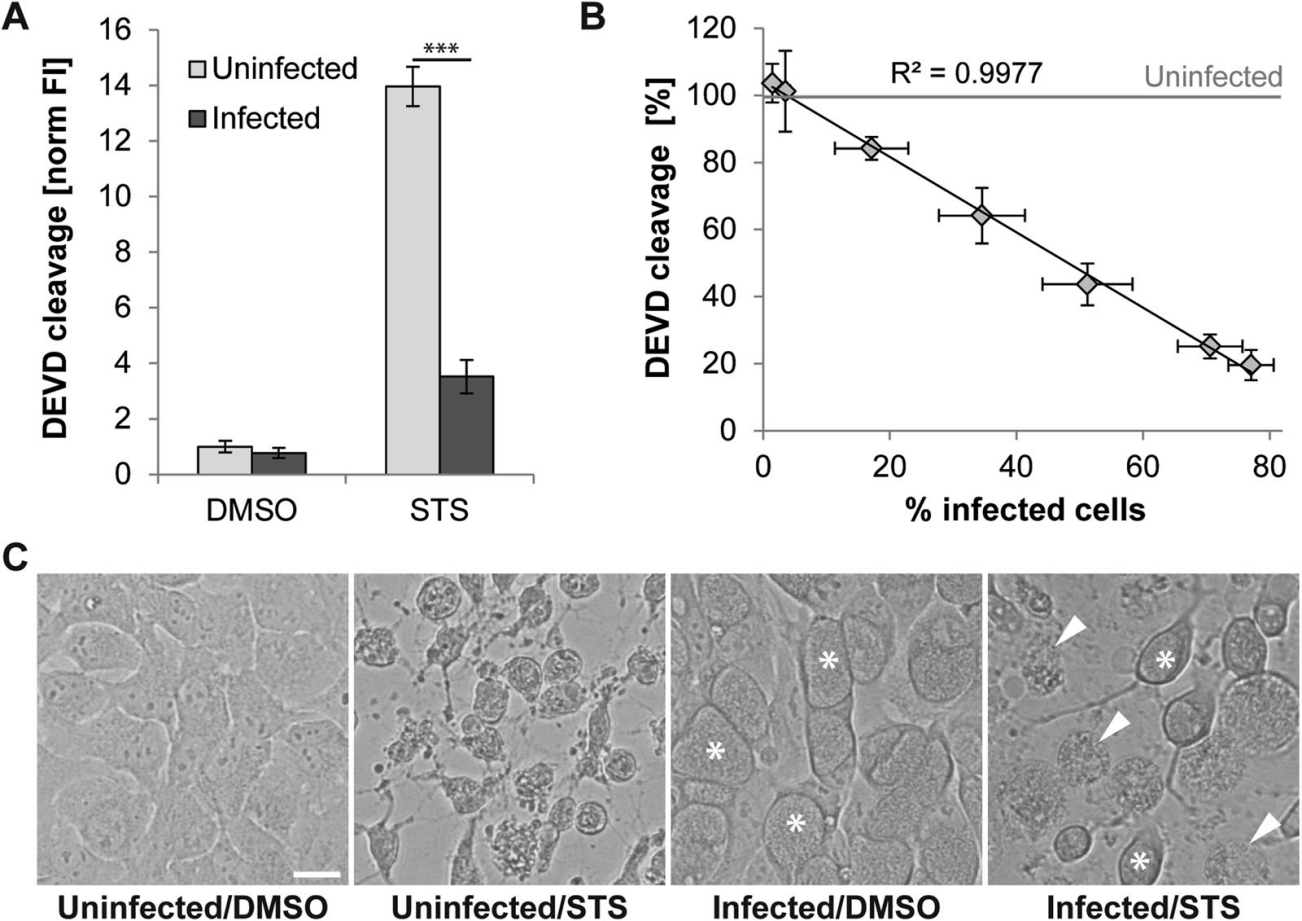
STS fails to induce apoptotic effector caspase activity in *C. trachomatis*-infected HeLa cells, but causes host cell lysis. **a** *C. trachomatis* inhibits induction of DEVD cleavage activity. HeLa cells infected with *C. trachomatis* (20 IFU/cell, 24 h) and uninfected control cells were treated with DMSO or STS (1.8 µM). DEVD cleavage activity in cell lysates was measured at 7 hpt and was normalized to the activity detected in uninfected DMSO-treated cells (mean ± SD, *n* = 4, ANOVA). **b** The strength of DEVD cleavage inhibition correlates with the level of infection. HeLa cells were infected with different doses of *C. trachomatis* and were treated with STS (1.8 µM) at 24 hpi. DEVD cleavage activity in cell lysates was measured at 7 hpt, normalized to the activity detected in STS-treated uninfected cells, and plotted against the percentage of infected cells determined microscopically from parallel cultures (mean ± SD, *n* = 3). **c** Phase contrast microscopy reveals signs of necrotic cell death in *C. trachomatis*-infected (10 IFU/cell) HeLa cells that were treated at 24 hpi for 7 h with STS (1 µM). Asterisks and arrowheads indicate examples of bacterial inclusions in apparently intact infected cells and examples of necrotic infected cells, respectively (scale bar, 20 µm)

While elaborating a strategy to identify *Chlamydia* factors that contribute to the inhibition of apoptosis, we monitored DEVD cleavage as a simple read-out for apoptotic effector caspase (CASP3/CASP7) activity [19]. Consistent with *C. trachomatis*’ reported anti-apoptotic activity [10, 12–14, 20], we observed that treatment of *C. trachomatis*-infected HeLa cells with STS failed to induce DEVD cleavage activity (Fig. 1a). Moreover, the decrease in DEVD cleavage correlated with the percentage of infected cells (Fig. 1b). Unexpectedly, microscopic inspection of STS-treated cultures indicated widespread induction of necrotic death in infected cells (Fig. 1c).

### Time-lapse microscopic analysis of infected cells confirms the engagement of necrotic death upon exposure to pro-apoptotic stimuli

**Fig. 2:**
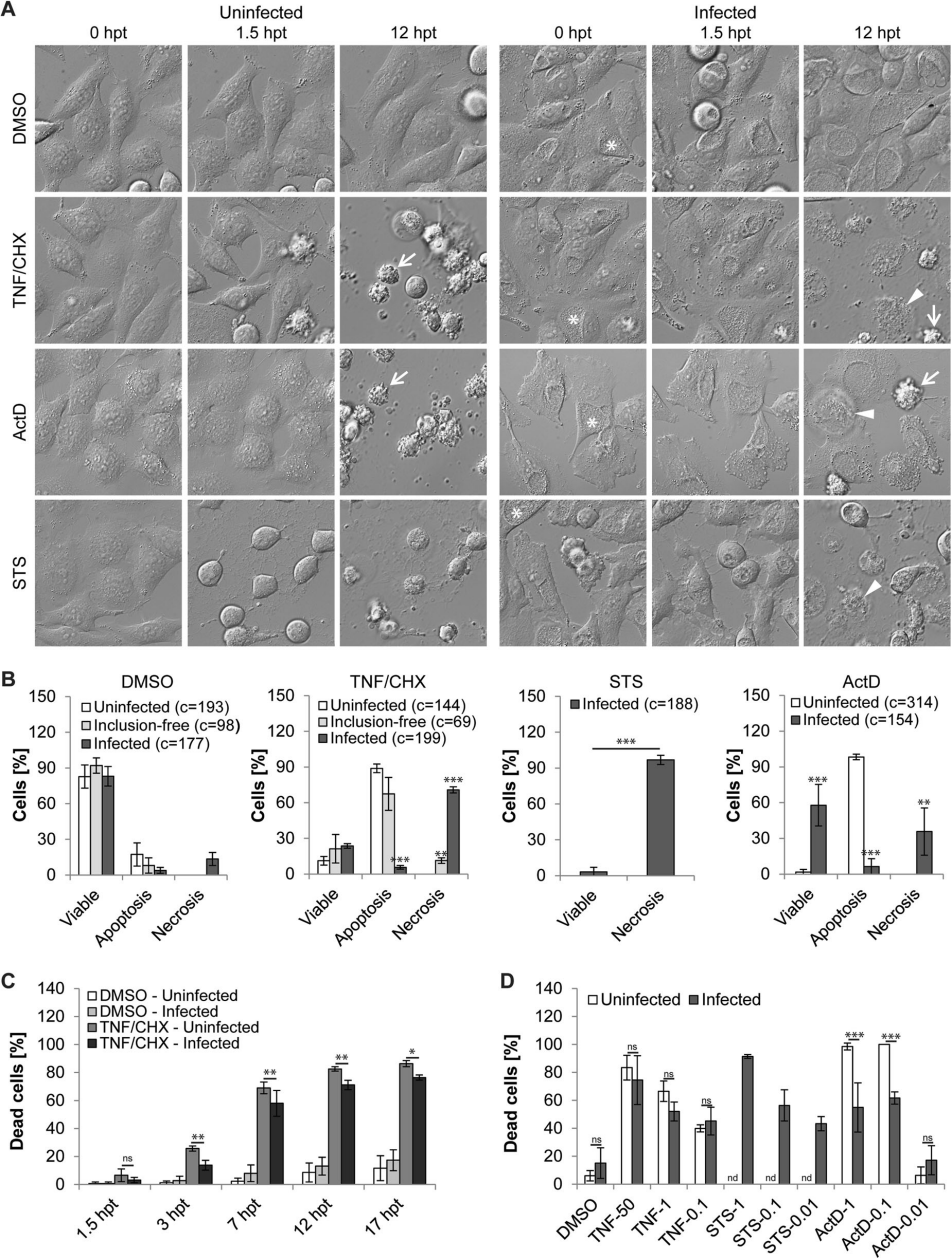
Time-lapse video microscopy confirms that apoptosis inducers stimulate necrotic death in *C. trachomatis*-infected HeLa cells. **a**–**c** Apoptosis inducers trigger necrotic cell death in inclusion-bearing cells. Uninfected and *Chlamydia*-infected (5 IFU/cell) HeLa cells were treated with apoptosis inducers (TNF-α (50 ng/ml + 2.5 µg/ml CHX), ActD (1 µM), or STS (1.3 µM); added at 24 hpi) and monitored by time-lapse microscopy until 17 hpt. (**a**) Selected images from time-lapse movies (movies S1-S4). Asterisks, arrowheads, and arrows indicate examples of inclusions, necrotic cells and apoptotic cells, respectively. **(b**) Quantitative assessment of the frequency of apoptosis and necrosis until 17 hpt (mean ± SD, *n* = 3, ANOVA (DMSO, TNF/CHX, ActD), *t*-test (STS); if not indicated else for each viability group significant differences compared to uninfected cells are marked). The category “uninfected” refers to cells in uninfected cultures, whereas the categories “inclusion-free” and “infected” refer to inclusion-free and inclusion-bearing cells in infected cultures. The total number of cells (c) analyzed in each group is indicated in the figure. (**c**) Comparison of the frequency of dying/dead cells (necrotic + apoptotic) among uninfected and infected cells at different time points post addition of TNF/CHX (mean ± SD, *n* = 3, ANOVA). **d** Weak pro-apoptotic stimulation is sufficient to induce necrosis in infected cells. Time-lapse microscopy-based quantitative assessment of cell death (necrotic + apoptotic) in infected (5 IFU/cell) and uninfected cells exposed for 17 h to various concentrations of apoptosis inducers (TNF-α, 50 ng/ml, 1 ng/ml, 0.1 ng/ml (+2 µg/ml CHX); STS, 1 µM, 0.1 µM, 0.01 µM; ActD, 1 µM, 0.1 µM, 0.01 µM). The category “uninfected” refers to cells in uninfected cultures, whereas the category “infected” refer to inclusion-bearing cells in infected cultures (mean ± SD, *n* = 4 (DMSO, TNF-50, TNF-1), *n* = 3 (all other groups), ANOVA; nd, not determined). The total number of cells analyzed for each group was ≥ 145

To assess the extent and mode of death induced by pro-apoptotic stimuli in *Chlamydia*-infected cells, we monitored live infected cultures by time-lapse video microscopy. We based our quantitative assessments on characteristic morphological hallmarks of apoptosis and necrosis (Fig. S1A). We first analyzed the effect of classical inducers of apoptosis, including TNF-α (50 ng/ml; added together with 2.5 µg/ml cycloheximide (CHX)), actinomycin D (ActD, 1 µM), and STS (1.3 µM), on uninfected HeLa cells. Most mock- (DMSO-) treated cells displayed normal adherent morphology, only sporadically interrupted by mitosis, throughout the period monitored (until 17 h post treatment (hpt)) (Fig. 2a, b, movie S1). In contrast, a large proportion of cells treated with TNF/CHX or ActD developed a typical apoptotic morphology, including cellular shrinkage, membrane blebbing, and detachment, followed eventually by secondary necrosis (Fig. 2a, b, movies S2-3). Cells treated with STS displayed drastic changes in morphology, such as a pronounced cell shrinkage [21], within minutes after addition of the drug (Fig. 2a, movie S4). These early effects hindered a quantitative analysis of apoptotic traits in STS-treated cells by time-lapse microscopy.

We next analyzed *C. trachomatis-*infected cells that were treated with apoptosis inducers at 24 h post infection (hpi), a time point at which *Chlamydia*-mediated inhibition of apoptosis is robust [10, 13] and inclusions were large enough to distinguish inclusion-bearing from inclusion-free cells by microscopy (Fig. S1B). The majority of infected DMSO-treated cells maintained a normal morphology and bacterial replication and inclusion expansion were readily detectable (Fig. 2a, b, movie S1). In the presence of TNF/CHX, inclusion-bearing cells predominately died by necrosis, displaying a sudden rupture of the host cell membrane in absence of morphological signs of apoptosis, while neighboring inclusion-free cells died by apoptosis (Fig. 2a, b, movie S2). The death of infected cells was not considerably delayed compared to the death of uninfected cells (Fig. 2c). Consistent with our initial observations, STS-induced necrotic death in >90% of infected cells during the period monitored (Fig. 2a, b, movie S4). Interestingly, infected cells appeared to be partially protected from cell death mediated by ActD, although this drug also induced necrotic death in a significant proportion of infected cells (Fig. 2a, b, movie S3).

### Weak pro-apoptotic stimulation is sufficient to induce necrotic death in infected cells

We next studied how infected cells would respond to weaker stimulation. Live cell monitoring of uninfected HeLa cells indicated that the percentage of cells displaying apoptotic features in response to TNF/CHX decreased as we lowered the concentration of TNF-α (Fig. 2d). Similarly, the percentage of infected cells undergoing necrosis diminished with decreasing concentrations of TNF-α, yet the overall percentage of cells that succumbed to TNF/CHX treatment was similar between infected and uninfected cultures at each concentration tested (Fig. 2d). A similar analysis could not be made for STS, because the onset of morphological features of apoptosis in STS-treated cells could not be assessed unequivocally. However, necrotic death of infected cells was readily observed when the STS concentration was reduced to one-tenth or one-hundredth of the initially tested standard dose (Fig. 2d).

### The induction of necrosis in response to pro-apoptotic stimuli is not cell line specific

**Fig. 3:**
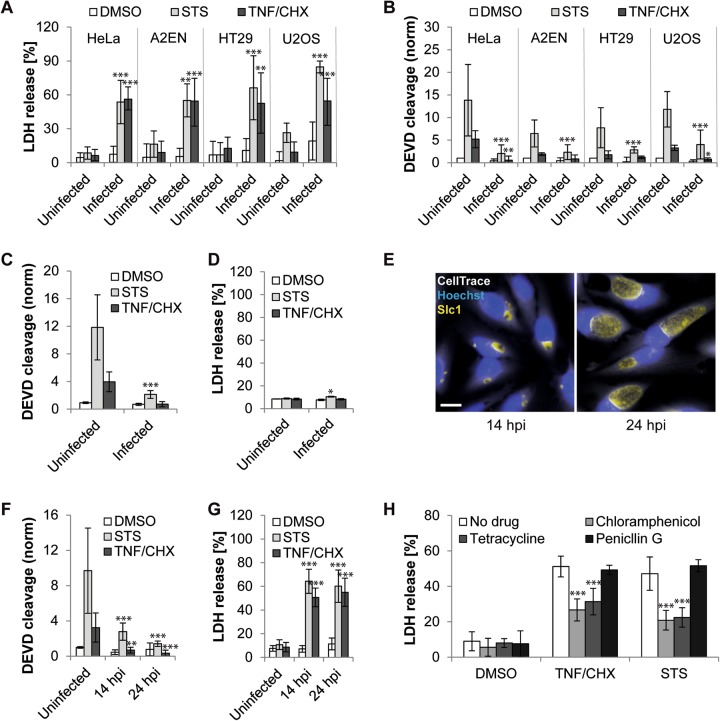
The *Chlamydia*-mediated shift from apoptosis to necrosis occurs in multiple cell lines and is partially dependent on a bacterial activity. **a**–**b** *C. trachomatis* shifts apoptosis to necrosis in multiple human cell lines. The graphs display early release of LDH (**a**) and reduced induction of DEVD cleavage (**b**) from/in infected (10 IFU/cell) cultures treated with pro-apoptotic drugs (STS (1 µM) or TNF-α (50 ng/ml (HeLa, U2OS) or 200 ng/ml (HT29, A2EN) + 2.5 µg/ml CHX); added at 24 hpi). Culture supernatants and cell lysates were collected/prepared at 7 hpt (HeLa) or 9 hpt (other cell lines) for measurement of LDH activity (**a**) and DEVD cleavage activity (**b**), respectively (mean ± SD, *n* = 3 (DEVD (all cell lines), LDH (U2OS)), *n* = 4 (LDH (other cell lines), ANOVA). **c**–**d** *C. trachomatis* blocks the induction of DEVD cleavage activity at a time point preceding necrotic cell death. HeLa cells were treated as described for (**a**, **b**). Culture supernatants and cell lysates were collected/prepared at 4 hpt for measurement of DEVD cleavage activity (**c**) and LDH activity (**d**), respectively (mean ± SD, *n* = 3, ANOVA). **e** Representative images displaying the difference in inclusion size in HeLa cell cultures infected with *Chlamydia* (10 IFU/cell) for 14 h or 24 h (Hoechst, blue; CellTrace CFSE, white; Slc1 (*Chlamydia*), yellow; scale bar, 20 µm). **f**, **g** Reduced induction of DEVD cleavage activity (**f**) and enhanced release of LDH (**g**) in/from HeLa cultures treated with pro-apoptotic drugs at an early stage of infection. Cells were treated with apoptosis inducers (as described for (**a**, **b**)) at 14 hpi or 24 hpi (10 IFU/cell). DEVD cleavage activity in cell lysates (**f**) and LDH activity in culture supernatants (**g**) were measured at 9 hpt (mean ± SD, *n* = 3, ANOVA). **h** Inhibitors of bacterial protein synthesis partially block necrotic death of infected (10 IFU/cell) HeLa cells that were exposed to apoptosis inducers (STS (1 µM) or TNF-α (50 ng/ml + 2.5 µg/ml CHX); added at 24 hpi). Antibiotics (chloramphenicol (1.5 µg/ml), tetracycline (2 µg/ml), and penicillin G (1 U/ml)) were added prior to the addition of apoptosis inducers. LDH activity in culture supernatants was measured at 9 hpt (mean ± SD, *n* = 6 (no drug, chloramphenicol), *n* = 3 (other groups), ANOVA). Statistically significant differences marked in Fig. 3 relate to differences (within each treatment group) in relation to uninfected cells (**a**–**d**, **f**, **g**) or the no drug control (**h**)

Although apoptotic cells will eventually lyse in cell culture, the integrity of their plasma membrane is not compromised during the initial stages of apoptosis [22]. Indeed, 7 h of exposure to TNF/CHX or STS did not cause significant release of the host enzyme lactate dehydrogenase (LDH) from uninfected HeLa cells, while LDH activity was readily detected in the supernatant of treated infected cultures (Fig. 3a). By conducting parallel measurements of DEVD cleavage activity in cell lysates, we further confirmed that TNF/CHX and STS induced apoptotic effector caspase activity within 7 h in uninfected cells, and that this activation was significantly reduced in infected cultures (Fig. 3b). Similar results were obtained when caspase activity was assessed at 4 hpt (Fig. 3c), a time point preceding necrotic death of infected cells (Fig. 3d).

To distinguish necrotic from apoptotic death we also used the Annexin V/propidium iodide (PI) assay [23], in which staining of intact cells with fluorescently labeled Annexin V indicates externalization of phosphatidylserine, a hallmark of apoptosis, whereas staining with the membrane-impermeable DNA dye PI indicates loss of plasma membrane integrity, a sign of necrosis. While most dying cells in uninfected TNF/CHX-treated cultures stained positive for Annexin V but not PI, most dying cells in infected cultures stained double positive (Fig. S2), indicating that uninfected cells died by apoptosis, while infected cells died by necrosis.

We next determined whether the shift from apoptosis to necrosis also occurs in other cell lines. We tested three additional human epithelial cell lines, including A2EN (endocervix), U2OS (bone osteosarcoma), and HT29 (colorectal adenocarcinoma) cells. TNF/CHX and STS added to infected cells induced necrotic death in all tested cell lines (Fig. 3a). In contrast, in uninfected cells, treatment resulted in most instances in the induction of DEVD cleavage activity (Fig. 3b).

### TNF/CHX- and STS-induced necrosis of *Chlamydia*-infected cells partially depends on bacterial activity

We considered the possibility that large inclusions could favor necrosis of infected cells under pro-apoptotic conditions due to mechanical stress. We thus tested how infected cells would respond to pro-apoptotic stimuli at 14 hpi (i.e., 10 h earlier than in previous experiments), a time point at which *Chlamydia* inclusions were still relatively small (Fig. 3e). While we observed that the block in the induction of DEVD cleavage activity was slightly weaker when pro-apoptotic drugs were added this early (Fig. 3f), STS and TNF/CHX induced similar extents of necrotic death regardless of the time point of treatment (Fig. 3g).

Our earlier observation that ActD, compared to other pro-apoptotic drugs tested, induced less necrosis in infected cells (Fig. 2b, d), suggested that this inhibitor of transcription may block a host or bacterial activity required for the execution of necrotic cell death. Because necrosis readily occurred in the presence of CHX, an inhibitor of host protein synthesis, we hypothesized that an activity exerted by the bacteria may be required. Indeed, antibiotics that block bacterial protein synthesis (chloramphenicol and tetracycline) partially reduced necrosis of infected cells when added shortly before exposure to STS or TNF/CHX, while penicillin G (which does not affect bacterial protein synthesis) had no protective effect (Fig. 3h).

### TNF/CHX-induced necrosis in *Chlamydia*-infected cells is not canonical necroptosis, but requires caspase activity

**Fig. 4:**
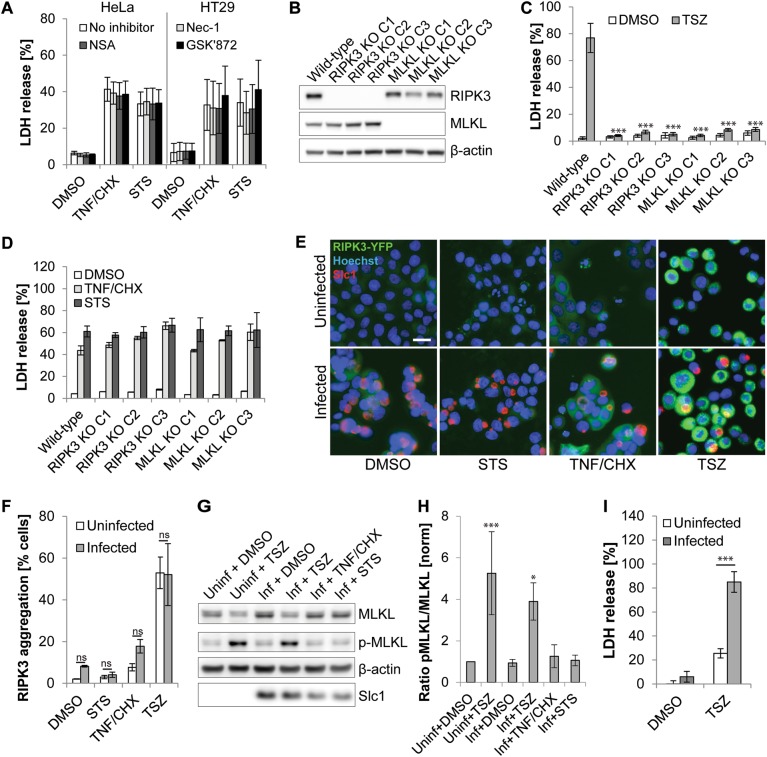
TNF/CHX-induced necrosis in *Chlamydia*-infected cells does not represent necroptosis. **a** Inhibitors of necroptosis fail to block necrotic death of infected (10 IFU/cell) HeLa or HT29 cells that were treated with apoptosis inducers (STS (1 µM) or TNF-α (50 ng/ml + 2.5 µg/ml CHX); added at 24 hpi). Inhibitors (GSK′872 (8 µM), necrosulfonamide (NSA, 2 µM), and necrostatin-1 (Nec-1, 40 µM)) were added prior to induction of cell death. LDH activity in culture supernatants was measured at 9 hpt (mean ± SD, *n* = 3, ANOVA; no statistically significant differences in relation to no inhibitor controls were observed). **b** Western blot analysis confirming the presence or absence of MLKL and RIPK3 in wild type, MLKL-deficient, and RIPK3-deficient HT29 cell lines. The designation C1-C3 refers to three distinct clonal cell populations obtained after selection of transduced cells. **c** MLKL- and RIPK3-deficient HT29 cells are protected from TSZ-induced necroptosis. HT29 cells with indicated gene deficiencies were treated with TSZ (a mixture of TNF-α (20 ng/ml), Smac mimetic BV6 (1 µM), and Z-VAD-FMK (50 µM)). LDH activity in culture supernatants was measured at 14 hpt (mean ± SD, *n* = 3, ANOVA). **d** Infected MLKL- or RIPK3-deficient HT29 cells are not protected from necrotic death induced by apoptosis inducers. HT29 cells with indicated gene deficiencies were infected (10 IFU/cell). At 24 hpi cells were treated with STS (1 µM) or TNF-α (200 ng/ml + 2.5 µg/ml CHX). LDH activity in culture supernatants was measured at 9 hpt (mean ± SD, *n* = 3, ANOVA; no statistically significant differences compared to wild-type control cells were observed). **e**, **f** Apoptosis inducers fail to induce RIPK3 activation in *Chlamydia*-infected cells. Infected (10 IFU/cell, 24 h) and uninfected HT29 cells expressing RIPK3-YFP were treated with TSZ (see **(c)**), STS (1 µM), or TNF-α (200 ng/ml + 2.5 µg/ml CHX). At 9 hpt cells were fixed, stained, and imaged (RIPK3-YFP, green; Hoechst, blue; *Chlamydia* (Slc1), red; scale bar, 20 µm) (**e**). The percentage of RIPK3-aggregation-positive cells (**f**) was determined based on a fluorescence intensity threshold that was inferred from the observed intensity in the controls (untreated and TSZ-treated uninfected cells) (mean ± SD, *n* = 3, ANOVA). **g**, **h** Apoptosis-inducing conditions fail to induce MLKL activation (phosphorylation) in *Chlamydia*-infected cells. Infected (10 IFU/cell, 24 h) and uninfected HT29 cells were treated with TSZ (see (**c**)), STS (1 µM), or TNF-α (200 ng/ml + 2.5 µg/ml CHX). At 7 hpt protein samples were generated for western blot analysis. Representative blot images from one of three independent experiments are shown in (**g**). A quantitative analysis of MLKL phosphorylation is shown in (**h**) (mean ± SD, *n* = 3, ANOVA). **i** *C. trachomatis* fails to block necroptosis. Infected (10 IFU/cell, 24 h) and uninfected HT29 cells were treated with TSZ (see (**c**)). LDH activity in culture supernatants was measured at 9 hpt (mean ± SD, *n* = 3, ANOVA)

When the apoptotic machinery is blocked, pro-apoptotic signals, such as TNF/CHX and STS, induce a form of regulated necrosis known as necroptosis [24, 25]. We thus tested the effect of inhibitors of the necroptotic pathway, including necrostatin-1 (inhibitor of RIPK1) [26], GSK’872 (inhibitor of RIPK3) [27], and necrosulfonamide (inhibitor of MLKL) [28], on death induced in infected cells. All three inhibitors blocked death in HT29 cells in which necroptosis was induced by addition of TNF-α in the presence of the Smac mimetic BV6 and the pan caspase inhibitor Z-VAD-FMK (a drug combination referred to as TSZ [29]) (Fig. S3). However, these inhibitors did not reduce necrosis in *Chlamydia*-infected HeLa or HT29 cells treated with apoptosis inducers (Fig. 4a). Consistent with these findings, RIPK3-deficient and MLKL-deficient HT29 cells were protected from TSZ-induced necroptosis (Fig. 4b, c), but not from necrotic death induced in infected cells upon exposure to pro-apoptotic drugs (Fig. 4d).

We further tested more directly for the activation of the necroptotic signaling pathway. In a HT29 biosensor cell line that expresses RIPK3-YFP, induction of necroptosis with TSZ led to RIPK3-YFP-aggregation, a hallmark of its activation, and elevated YFP fluorescence intensity (Fig. 4e, f). RIPK3 activation was neither observed in DMSO-treated infected cells nor was it induced when infected cells were exposed to pro-apoptotic drugs (Fig. 4e, f). A time course analysis indicated that RIPK3 activation was also not detectable at earlier time points after stimulation with STS or TNF/CHX (Fig. S4). Similarly, phosphorylation of MLKL, an indicator for MLKL activation, was not observed in infected HT29 cells that were treated with DMSO or apoptosis inducers (Fig. 4g, h). Interestingly, these experiments also revealed that infection with *Chlamydia* did not prevent RIPK3 or MLKL activation induced by TSZ in HT29 cells (Fig. 4e–h) and rather enhanced resulting necrotic cell death (Fig. 4i).

**Fig. 5:**
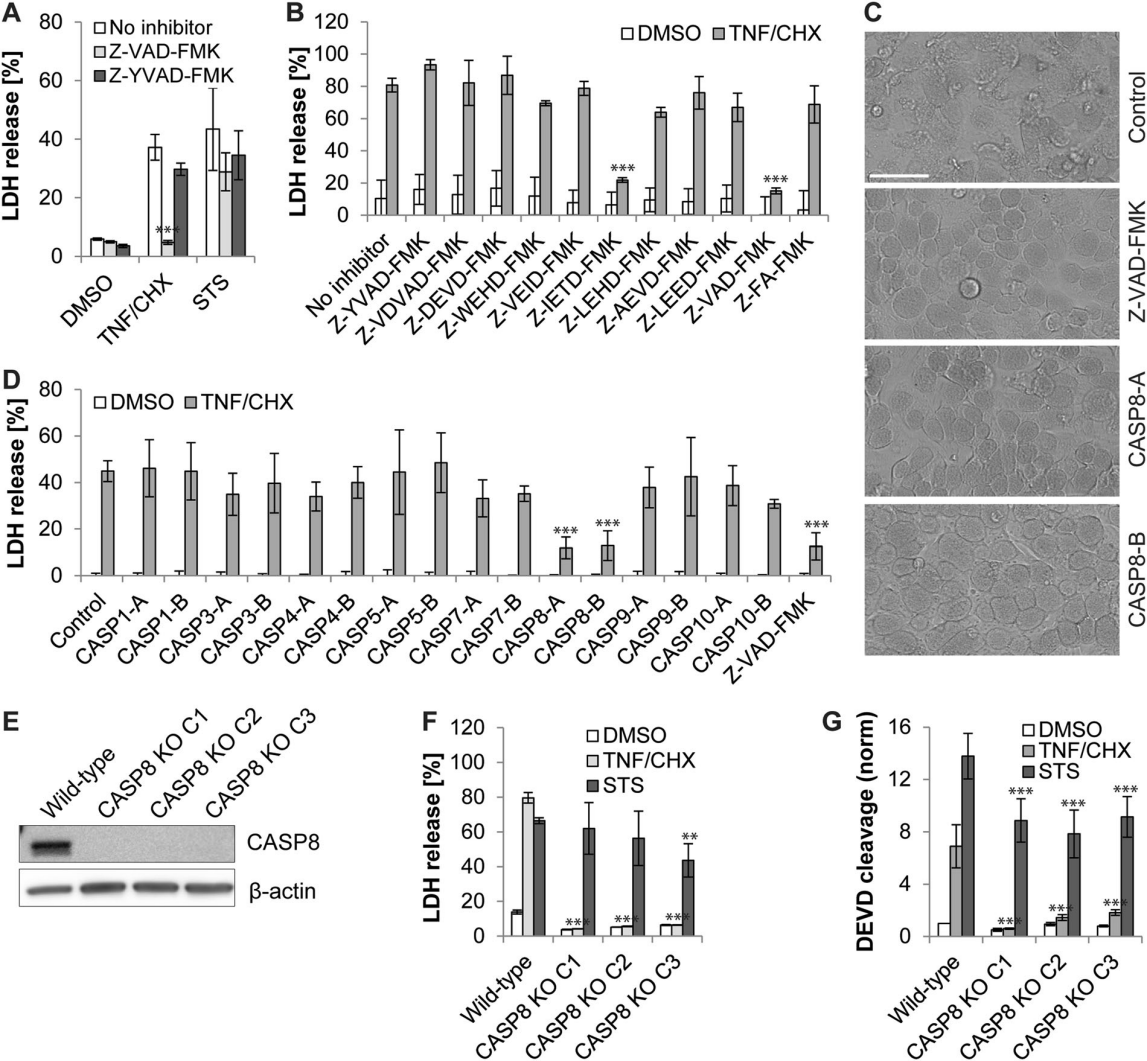
TNF/CHX induces CASP8-dependent necrosis in *Chlamydia*-infected cells. **a** A pan caspase inhibitor blocks TNF/CHX-induced necrotic death of infected (10 IFU/cell) HeLa cells. Apoptosis inducers (STS (1 µM) or TNF-α (50 ng/ml + 2.5 µg/ml CHX)) were added at 24 hpi. Caspase inhibitors (Z-VAD-FMK (40 µM), Z-YVAD-FMK (10 µM)) were added prior to induction of cell death. LDH activity in culture supernatants was measured at 9 hpt (mean ± SD, *n* = 3, ANOVA). **b** Inhibition of CASP8 blocks TNF/CHX-induced necrotic death of infected (10 IFU/cell) HeLa cells. TNF-α (50 ng/ml + 2.5 µg/ml CHX) was added at 24 hpi. Inhibitors from Enzo’s caspase inhibitor set IV (each at 10 µM) were added prior to induction of cell death. LDH activity in culture supernatants was measured at 9 hpt (mean ± SD, *n* = 3, ANOVA). **c**, **d** Depletion of CASP8 blocks TNF/CHX-induced necrotic death of infected HeLa cells. Cells were infected (10 IFU/cell) 18 h after transfection with specific siRNAs. For each target 2 distinct siRNAs were tested. TNF-α (50 ng/ml + 2.5 µg/ml CHX) was added at 24 hpi. Phase contrast images were made prior to the collection of supernatants (scale bar, 40 µM) (**c**). LDH activity in culture supernatants was measured at 9 hpt (mean ± SD, *n* = 6 (control, Casp3-A/B, Casp4-A/B, Casp8-A/B, Casp9-A/B), *n* = 3 (other groups), ANOVA) (**d**). **e** Western blot analysis displaying the presence or absence of CASP8 in wild-type and CASP8-deficient HeLa cells. The designation C1-C3 refers to three distinct clonal cell populations obtained after selection of transduced cells. **f** CASP8-deficient infected (10 IFU/cell, 24 h) HeLa cells are protected from TNF/CHX-induced necrosis. Cells were treated with STS (1 µM) or TNF-α (50 ng/ml + 2.5 µg/ml CHX). LDH activity in culture supernatants was measured at 9 hpt (mean ± SD, *n* = 3, ANOVA). **g** Uninfected CASP8-deficient HeLa cells are protected from TNF/CHX-induced, but not STS-induced, apoptosis. Cells were treated with STS (1 µM) or TNF-α (50 ng/ml + 2.5 µg/ml CHX). DEVD cleavage activity in cell lysates was measured at 7 hpt and was normalized to the activity detected in DMSO-treated cells (mean ± SD, *n* = 3, ANOVA). Statistically significant differences marked in Fig. 5 relate to differences in relation to no inhibitor/no depletion controls (a-b,d) or wild-type cells (f-g)

After excluding a major role for the canonical necroptotic cell death pathway in TNF/CHX- and STS-induced necrosis of infected cells, we next tested for a potential involvement of CASP1-mediated pyroptosis [30]. We observed that the CASP1 inhibitor Z-YVAD-FMK, like the necroptosis inhibitors, failed to significantly affect the induction of necrotic death in infected TNF/CHX- or STS-treated HeLa cells (Fig. 5a). Interestingly, the pan caspase inhibitor Z-VAD-FMK strongly reduced LDH release from infected cultures that were exposed to TNF/CHX, while its effect on STS-induced death was only minor (Fig. 5a). By testing a panel of caspase-specific inhibitors, we determined that TNF/CHX-induced necrosis in infected cells could be blocked by Z-IETD-FMK, an inhibitor of CASP8 (Fig. 5b). CASP8 is an apoptotic initiator caspase that acts downstream of the TNF-α receptor in the canonical TNF/CHX-mediated apoptosis pathway [31], but only plays a minor role in STS-induced apoptosis [32]. Depletion of CASP8 with specific siRNAs protected infected cells from TNF/CHX-induced necrosis (Fig. 5c, d). Consistent with these findings, infected CASP8-deficient HeLa cells were protected from TNF/CHX-induced necrotic death (Fig. 5e, f), while uninfected CASP8-deficient cells were protected from TNF/CHX-induced apoptosis (Fig. 5g). Together these data indicate that, unlike the activation of apoptotic effector caspases (Fig. 3b), the early steps in TNF/CHX-induced apoptosis leading to CASP8 activation are not blocked in cells infected with *C. trachomatis* and participate in TNF/CHX-induced necrosis of infected cells. Consistent with this notion, western blot analysis confirmed that CASP8, but not CASP3, was processed into its active form in infected TNF/CHX-treated cells (Fig. S5).

### Apoptosis inducers perturb *Chlamydia* development

**Fig. 6:**
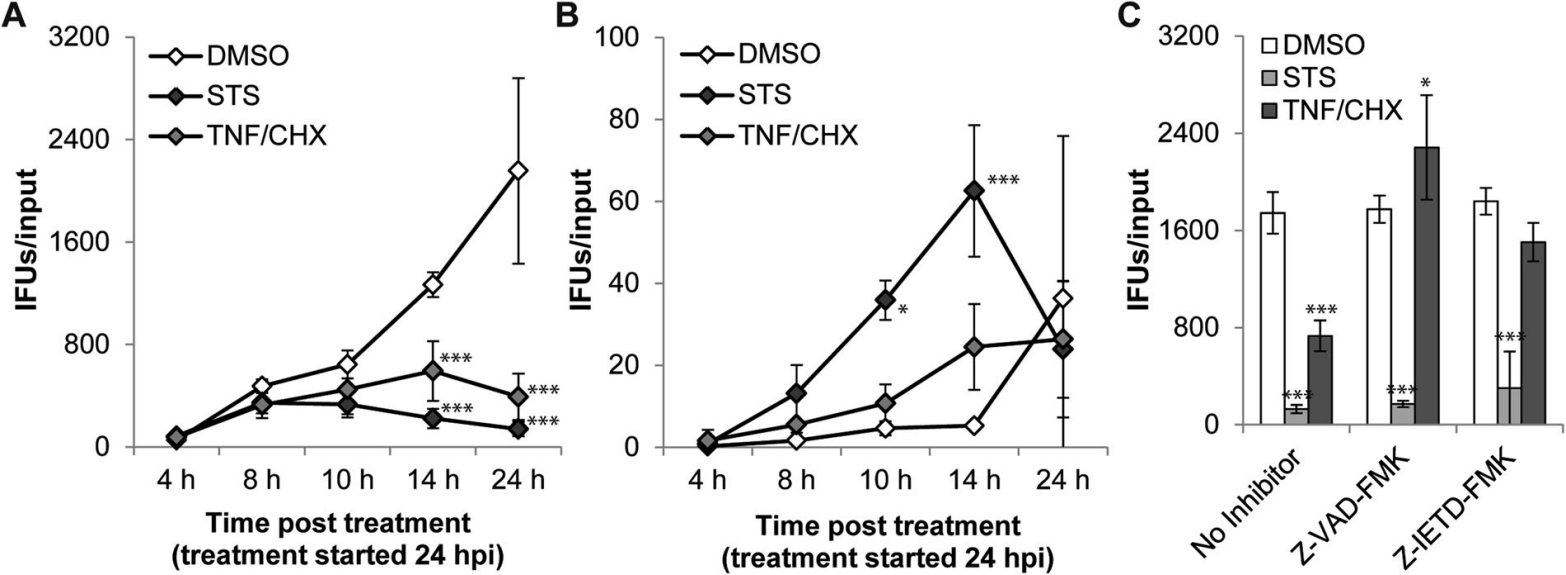
Pro-apoptotic conditions disrupt *Chlamydia* development. **a**, **b** Quantification of IFU production in *C. trachomatis*-infected HeLa cells exposed to pro-apoptotic stimuli. Confluent monolayers of HeLa cells were infected with *C. trachomatis* and treated with apoptosis inducers (STS (1 µM) or TNF-α (50 ng/ml + 2.5 µg/ml CHX)) at 24 hpi. Cell lysates and culture supernatants were prepared/collected at indicated time points and the number of IFUs was quantified and normalized to the input IFUs used for the initial infection. Displayed data represent total numbers of IFUs (lysates + supernatants) (**a**) and numbers of IFUs in supernatants (**b**), respectively (mean ± SD, *n* = 3, ANOVA; significant differences compared to the DMSO control are displayed for each time point). **c** Inhibition of CASP8 restores production of infectious progeny in presence of TNF/CHX. HeLa cells were treated as described for (**a**, **b**), yet caspase inhibitors (Z-VAD-FMK (10 µM), Z-IETD-FMK (10 µM)) were added prior to the addition of pro-apoptotic drugs. Displayed data represent IFUs in cell lysates prepared at 24 hpt (mean ± SD, *n* = 3, ANOVA; significant differences compared to the DMSO control are displayed for each inhibitor group)

The inhibition of apoptosis by *Chlamydia* spp. is commonly interpreted as a means to maintain host cell integrity to enable continued bacterial replication [9, 10, 33]. Yet, our findings suggest that *C. trachomatis*-infected cells undergo necrosis under pro-apoptotic conditions. We thus tested the effect of apoptosis inducers on the production of infectious EBs—a hallmark of a completed infectious cycle. For this purpose, HeLa cells infected with *C. trachomatis* were treated with apoptosis inducers at 24 hpi and the number of infectious particles released into the culture supernatant or contained in remaining cells was quantified at different time points post treatment. During normal development, *C. trachomatis* L2 RBs start to differentiate into infectious EBs at around mid-stage of the infection cycle (about 20–24 hpi), yet host cell integrity is maintained until late stages (about 40–48 hpi) when EBs are eventually released by host cell lysis [6, 34]. Indeed, in DMSO-treated control cultures, the overall number of infectious particles produced until 28 hpi was low, but increased continuously thereafter (Fig. 6a). Moreover, until 38 hpi no significant release of infectious particles from infected cells was detected (Fig. 6b). Consistent with the induction of necrotic host cell death and hence a loss of host plasma membrane integrity, STS and TNF/CHX added at 24 hpi induced the release of a small amount of infectious bacterial particles (Fig. 6b). Yet, the premature loss of the replicative niche abolished further production of infectious bacteria (Fig. 6a). Importantly, inhibition of necrotic host cell death with Z-VAD-FMK or Z-IETD-FMK restored normal production of infectious bacteria in presence of TNF/CHX (Fig. 6c).

## Discussion

The anti-apoptotic effect of *C. trachomatis* was first recognized two decades ago [10] and described as being of “unusual strength and quality” when compared with similar activities exerted by other intracellular bacterial pathogens [35]. Given *Chlamydia*’s obligate intracellular nature [5], it was reasonable to conclude that one of the roles of assuming an anti-apoptotic state is to protect the host cell, the replicative niche, from cytotoxic insults and stress emanating from immune mediators or the infection itself [9, 33].

Here we report that the anti-apoptotic capacity of *C. trachomatis* is insufficient to protect the infected cell from death. We observed that under pro-apoptotic conditions, infected cells die by necrosis, a type of death that is characterized by a sudden rupture of the host cell plasma membrane (Figs 1c, 2a, b, and movies S2-S4) and release of host cell contents (Fig. 3a), but is not accompanied by the activation of apoptotic effector caspases (Figs 1a, b and 3b) or morphological hallmarks of apoptosis (Fig. 2a, b and movies S2-S4). It should be noted that this host cell death is premature and thus distinct from host cell lysis that naturally occurs at the end of the infection cycle. Indeed, the onset of necrotic death in infected cells that were exposed to pro-apoptotic stimuli occurred with similar kinetics as the manifestation of apoptotic morphologies in uninfected cultures (Fig. 2c), demonstrating that *C. trachomatis* does not prolong the lifespan of its host cell under these conditions. Consistent with this notion, the production of infectious *Chlamydia* particles was stunted by pro-apoptotic stimulation (Fig. 6a).

Previous studies that described the absence of hallmarks of apoptosis in *Chlamydia*-infected cells exposed to pro-apoptotic stimuli, focused on very early time points after treatment [10, 12–14, 20]. Cell death is a highly dynamic process. When exposed to a pro-death stimuli, individual cells in cultures die at different times, progress through different stages and can be lost for analysis as they detach or disintegrate [36]. Thus, short incubation periods may be optimal for the detection of apoptotic hallmarks. However, they do not allow monitoring the ultimate fate of these cells. While we did not conduct an exhaustive monitoring of all molecular hallmarks of apoptosis, our observation that infection blocked DEVD cleavage activity and externalization of phosphatidylserine (Fig. 3b, c and S2) is consistent with previously described anti-apoptotic activities ascribed to *Chlamydia* [10–14]. However, live cell imaging enabled us to monitor infected cells responding to pro-apoptotic stimuli over prolonged periods of time (Fig. 2), which uncovered an underappreciated complexity in *Chlamydia*’s ability to modulate programmed cell death. It should be noted that Jungas and coworkers previously noticed that, although *Chlamydia*-infected cells were protected from STS-induced apoptosis, the drug still caused a reduction in cell numbers in cultures of adherent infected cells [37]. Although the fate of the detached cells was not further characterized, these findings are consistent with our observation that *Chlamydia* fails to maintain host cell viability.

**Fig. 7:**
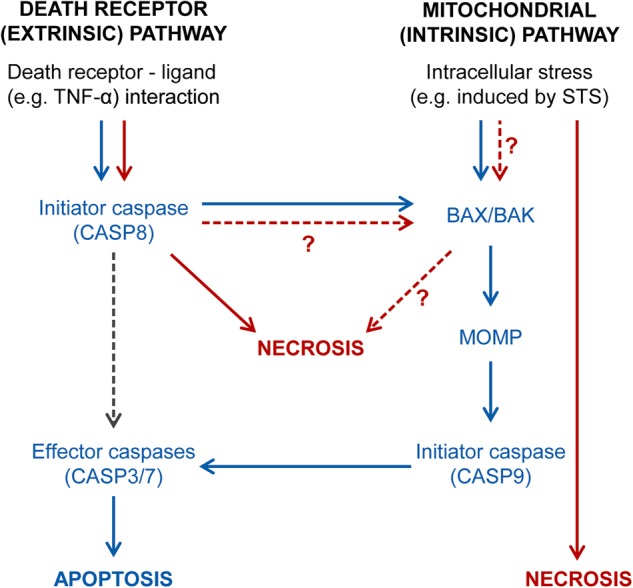
Model of pro-death signaling induced by pro-apoptotic conditions in uninfected cells or cells infected with *C. trachomatis*. Blue arrows indicate canonical signaling in the extrinsic and intrinsic pathways of apoptosis, as observed in uninfected cells. The gray dashed arrow indicates direct activation of effector caspases and apoptosis by CASP8 in a manner independent of MOMP (which only occurs in certain cell types [44]). Red arrows indicate deviations from canonical signaling observed in cells infected with *C. trachomatis*. It is currently unknown whether the pathways of TNF/CHX-induced and STS-induced necrosis in infected cells are distinct or converge (for instance at the level of BAX/BAK activation)

The pathways leading to necrotic cell death in *Chlamydia*-infected cells are non-canonical. The two major pro-apoptotic stimuli used in this study, STS and TNF/CHX, induce apoptosis via different routes of signaling, the intrinsic and the extrinsic pathway of apoptosis, respectively (Fig. 7). A central step in the intrinsic pathway is the mitochondrial outer membrane permeabilization (MOMP) [38], which is mediated by the pro-apoptotic proteins BAX and BAK [39] and leads to the release of cytochrome c and activation of the initiator caspase CASP9 [40]. CASP9 in turn activates the effector caspases (CASP3 and CASP7) that initiate the demolition of the cell [41, 42]. In the extrinsic pathway, engagement of death receptors, such as for instance the TNF-α receptor, can lead to the formation of a signaling complex that activates initiator caspase CASP8 [43]. While CASP8 can activate apoptotic effector caspases directly in some cell types, in most instances cell death induction requires an amplification of pro-death signaling via CASP8-dependent induction of MOMP [44]. When CASP8 is absent or inactivate, TNF-α induced signaling can induce RIPK1-RIPK3-MLKL-dependent canonical necroptosis as alternative mode of cell death [31]. Because conflicting observations have been reported on the effect of *Chlamydia* infection on CASP8 activation downstream of death receptor ligation [20, 45–47], we initially considered canonical necroptosis as a possible explanation for the death observed in TNF/CHX-treated infected cells. However, we observed that this cell death was not mediated by the RIPK3-RIPK1-MLKL machinery (Fig. 4a–h). Furthermore, we determined that CASP8 activation occurs in infected cells exposed to TNF/CHX (Fig. S5) and is required for the induction of necrotic cell death (Fig. 5).

Further work will be required to decipher how CASP8 induces this non-canonical form of necrotic cell death and if there is convergence between TNF/CHX- and STS-induced necrosis. For instance, both pathways may lead to activation of BAX and BAX-dependent caspase-independent modes of cell death were proposed to play a role in *Chlamydia* exit at the late stage of infection [7, 8, 48]. However, this scenario would need to be reconciled with the lack of BAX activation in response to pro-apoptotic stimuli in cells infected with *C. trachomatis* [11, 14, 37].

Why does *C. trachomatis* employ multiple anti-apoptotic strategies [15–18, 45, 49], if these are insufficient to keep the host cell alive? *Chlamydia* spp. induce necrotic host cell lysis as a natural mode of egress at the end of the infection cycle [6], although apoptotic morphological features have also been occasionally described in dying infected cells [7, 8]. Apoptotic cell death, if occurring sufficiently late in the infection cycle, may be a more favorable exit mechanism for the bacteria if it enables silent spread of infection by avoiding inflammatory responses often associated with necrosis [48]. However, *Chlamydia* spp. may not rely exclusively on this mechanism of spread, because they can also exit cells by extrusion [6]. During extrusion *Chlamydia* inclusions or parts of it are expelled from infected cells as vesicles that expose phosphatidylserine at their surface and may promote silent spread of infection, because the vesicles are taken up by neighboring cells or phagocytes in a manner analogous to the clearance of apoptotic bodies [6, 50]. It is possible that by blocking apoptotic death, the bacteria established necrosis as a second mode of egress to potentiate the number of cells that can be infected by the progeny emerging from a single infected cell. Alternatively, it is also possible that the anti-apoptotic state associated with infections with *Chlamydia* spp. has no specific benefit for the bacteria, but may be a secondary consequence of *Chlamydia*’s modulation of other host cell processes, such as the reprogramming of host cell metabolism.

Altogether our results suggest that the consequences of *C. trachomatis’* anti-apoptotic activities on the viability of infected host cells are more nuanced than initially thought and that the role of the modulation of apoptosis in *Chlamydia* pathogenesis remains to be determined.

## Methods and materials

### Cell culture

Vero (ATCC CCL-81), HeLa (ATCC CCL-2), HEK293T (ATCC CRL-3216), U2OS (ATCC HTB-96), and HT29 (ATCC HTB-38) cells, as well as RIPK3-YFP-expressing HT29 cells (stably transfected with a RIPK3-YFP expression plasmid [51] that was kindly provided by Zheng-Gang Liu), were grown in Dulbecco’s Modified Eagle’s Medium (DMEM; Thermo-Fisher-Scientific) supplemented with 10% heat-inactivated (56 °C, 30 min) fetal bovine serum (FBS; Thermo-Fisher-Scientific). DMEM devoid of phenol red was used for the analysis of host cell death to avoid interference of phenol red with photometric or fluorimetric assays. A2EN cells [52] were cultivated in Keratinocyte-SFM (Thermo-Fisher-Scientific) supplemented with 10% heat-inactivated FBS and 0.3 mM CaCl_2_. Cultures were shown to be free of *Mycoplasma* contamination by a PCR assay described elsewhere [53].

### Generation of knockout cells

Cell lines deficient for the indicated genes were generated using CRISPR/Cas9-mediated genome editing [54]. A lentiviral delivery system was used to introduce genes coding for Cas9 nuclease and gene-specific sgRNAs. The following sgRNAs, which were recently validated by Sanjana et al [55], were cloned into vector lentiCRISPRv2 [55] (a gift from Feng Zhang, Addgene): MLKL (5′-TTGAAGCATATTATCACCCT-3′, 5′-TTCTGAGAAGATCCGCAAGC-3′), RIPK3 (5′-CAGTGTTCCGGGCGCAACAT-3′, 5′-AACTGTTTGTTAACGTAAAC-3′), CASP8 (5′-TCCTTTGCGGAATGTAGTCC-3′, 5′-AGTCGTTGATTATCTTCAGC-3′). Lentiviral particles were harvested from supernatants of HEK293T cells that had been co-transfected with the packaging plasmid psPAX2 (a gift from Didier Trono, Addgene), the envelope plasmid pMD2.G (a gift from Didier Trono, Addgene), and the respective sgRNA-encoding derivative of lentiCRISPRv2. After filtration (0.45 µm) virus-containing supernatants were used to transduce HeLa or HT29 cells in the presence of 8 µg/ml polybrene (Sigma-Aldrich). Cells were co-infected with viruses encoding the two distinct sgRNAs (see above) that target the same gene. Transduced cells were selected in presence of puromycin (1 µg/ml (HeLa), 2 µg/ml (HT29); Thermo-Fisher-Scientific) and cloned by limiting dilution. Gene knock-outs were verified by western blot analysis (see below).

### Infection with *Chlamydia*

The majority of experiments described were made using the *C. trachomatis* wild-type strain L2/434/Bu (ATCC VR-902B). Only in the initial experiments (i.e., the testing of the DEVD cleavage assay (Fig. 1)) we used a rifampin–resistant variant, described elsewhere [56]. For the preparation of infection stocks, EBs were released from infected Vero cells by H_2_O-mediated lysis, purified by density gradient centrifugation, and stored at −80 °C in SPG buffer [57]. Bacteria were titered (determination of inclusion-forming units (IFUs)) and tested for *Mycoplasma* contamination as described previously [57]. For infection, cells were seeded in multi-well plates, followed by addition of bacteria (number of IFU/cell as specified), centrifugation (1500 × *g*, 30 min), and incubation for indicated periods of time. During the initial testing of the DEVD cleavage assay (Fig. 1) infections were made in suspension, i.e., cells and bacteria were mixed before seeding and centrifugation.

### Induction and inhibition of cell death

Apoptosis was induced by replacing the growth medium with medium containing STS, ActD, or human TNF-α added together with CHX (each purchased from Sigma-Aldrich) at the indicated concentrations. Necroptosis was induced by replacing the growth medium with medium containing TSZ (i.e., 20 ng/ml human TNF-α (Sigma-Aldrich), 1 µM Smac mimetic BV6 (Tocris), and 50 µM Z-VAD-FMK (Tocris)). In control wells, DMSO was added instead of pro-death drugs. Treated cells were incubated in presence of pro-death drugs until the end of the monitoring period (time-lapse microscopy) or the time point of analysis (all other experiments). When indicated, cell death inhibitors (necrosulfonamide (Tocris), necrostatin-1 (Tocris), GSK′872 (Merck), Z-VAD-FMK (Tocris), or inhibitors from the caspase inhibitor set IV (Enzo)) or antibiotics (chloramphenicol, tetracycline, and penicillin G (each purchased from Sigma-Aldrich)) were added at the indicated concentrations prior to cell death induction.

### Live cell phase contrast and time-lapse microscopy

Phase contrast images not derived from time-lapse microscopy experiments were made on an EVOS FL microscope (Thermo-Fisher-Scientific). Time-lapse microscopy experiments were conducted using two different instrumental set-ups. For the initial experiments testing single concentrations of pro-apoptotic drugs, cells were seeded and infected (5 IFU/cell) in glass bottom 6-well plates (In Vitro Scientific). At 22 hpi the medium was replaced with HEPES (10 mM)-buffered DMEM (without phenol red). At 24 hpi apoptosis inducers were added to respective wells, cells were placed on an atmospherically controlled stage (37 °C, 5% CO_2_) and imaged at 5–10 min intervals until 17 hpt on an Inverted Axio Observer.Z1 microscope (Zeiss). For later experiments testing variable concentrations of pro-apoptotic drugs, cells were seeded and infected (5 IFU/cell) in black clear-bottom 96-well plates (Greiner Bio One). At 24 hpi, apoptosis inducers were added to respective wells and cells were imaged at 12–15 min intervals until 17 hpt on an ImageXpress Micro XL system (Molecular Devices) (37 °C, 5% CO_2_). The software used to operate these systems (Metamorph 7.8 (Molecular Devices) and MetaXpress 5.3.0.4 (Molecular Devices), respectively) was also used to generate time-lapse movies. The fate of individual cells was determined by manual inspection of these movies. Cells that left the microscopic field during the period of imaging were excluded from the analysis. Daughters of dividing cells were considered as separate individual cells. The manual inspection was conducted by a researcher knowledgeable about the treatment group. However, a reanalysis of a randomly selected set of movies by a person blinded to treatment group and expected outcome gave virtually identical results.

### Immunofluorescence microscopy

Immunofluorescence staining of formaldehyde-fixed cells (2%, 20 min) in 96-well plates was conducted as described previously [57]. A rabbit-anti-Slc1 serum (1:400, described elsewhere [58]) in combination with AlexaFluor555- or AlexaFluor647-labeled secondary antibodies (Thermo-Fisher-Scientific) was used for the detection of *C. trachomatis*. When indicated, DNA was stained for 10 min with 2–10 µg/ml Hoechst 33342 (Thermo-Fisher-Scientific) and cells were stained for 10 min with either 5 µM CellTrace CFSE (Thermo-Fisher-Scientific) or 2 μg/ml HCS CellMask Deep Red stain (Thermo-Fisher-Scientific). Images were acquired on an ImageXpress Micro XL system (Molecular Devices) or on a Cellomics ArrayScan VTI HCS imaging system (Thermo-Fisher-Scientific). Automated image analysis (such as for the determination of the percentage of infected cells, inclusion numbers, or average fluorescence intensities per cell) was conducted using MetaXpress 5.3.0.4 (Molecular Devices) or HCS Studio Cell Analysis Software (Thermo-Fisher-Scientific), respectively.

### Annexin V/PI assay

The AlexaFluor488 Annexin V/Dead Cell Apoptosis Kit (Thermo-Fisher-Scientific) was adapted for use with adherent cells. Briefly, to each well in a 96-well plate containing treated or control cells in 50 µl growth medium, 50 µl of a 2x staining solution (PI (2 µg/ml) and Annexin V-AlexaFluor488 (1:10) in 2x binding buffer) were added. After 15 min incubation at room temperature, 100 µl 1x binding buffer containing 2 µg/ml Hoechst 33342 were added per well and cells were imaged on a Cellomics ArrayScan VTI HCS imaging system (Thermo-Fisher-Scientific). Fluorescence intensity thresholds for distinction between PI- and Annexin V-positive or negative cells were set so that about 95% of all cells in untreated uninfected control wells were classified as double negative (viable) cells.

### LDH release and DEVD cleavage

Quantification of LDH release as indicator for host cell lysis was conducted using the colorimetric in vitro toxicology assay kit from Sigma-Aldrich, as described recently [57]. LDH activity was normalized to the activity observed in total cell lysates, i.e., the maximum activity expected to be observed if 100% of the cells would lyse. Quantification of DEVD cleavage activity in cell lysates as indicator for the activity of apoptotic effector caspases (CASP3/CASP7) was conducted using the fluorimetric CASP3 assay kit from Sigma-Aldrich, as described recently [57]. Fluorescence intensity values were normalized to the mean fluorescence intensity observed for DMSO-treated uninfected cells. To display the correlation between infection and inhibition of DEVD cleavage, DEVD cleavage activity detected after infection with different doses of bacteria was normalized to the activity detected in STS-treated uninfected cells. Absorbance and fluorescence readings were made on an EnSpire 2300 (PerkinElmer), SpectraMax i3 (Molecular Devices), or Tecan infinite 200 (Tecan) plate reader.

### Depletion of caspases with siRNAs

HeLa cells were transfected with siRNAs according to transfection guidelines provided by Dharmacon. DharmaFECT-1 (Dharmacon) was used as transfection reagent; siRNAs were used at a concentration of 25 nM. To reduce toxicity, the transfection medium containing siRNAs and reagent was removed after 6 h incubation and replaced with fresh growth medium. Cells were incubated for additional 12 h prior to infection and further processing. In control transfections siRNA-free siRNA buffer (Dharmacon) was added to the transfection medium instead of siRNAs. For each target two different ON-TARGETplus siRNAs (purchased from Dharmacon) were used, including siRNAs targeting human CASP1 (A: GGAAGACUCAUUGAACAUA, B: GAUGGUAGAGCGCAGAUGC), CASP3 (A: CCGACAAG CUUGAAUUUAU, B: CCACAGCACCUGGUUAUUA), CASP4 (A: GGACUAUAGUGUAGAUGUA, B: CAACGUAUGGCAGGACAAA), CASP5 (A: CUACACUGUGGUUGACGAA, B: CCAUAGAACGAGCAACCUU), CASP7 (A: GGGCAAAUGCAUCAUAAUA, B: GAUCAGGGCUGUAUUGAAG), CASP8 (A: GGACAAAGUUUACCAAAUG, B: GCCCAAACUUCACAGCAUU), CASP9 (A: GAGUCAGGCUCUUCCUUUG, B: CGGUGAAAGGGAUUUAUAA), CASP10 (A: ACAAGGAAGCCGAGUCGUA, B: UGGCAGAACUCCUCUAUAU).

### Western blot analysis

For western blot analysis, protein extracts were generated by lysing cells in boiling 1% SDS buffer as previously described [57]. Proteins were mixed with loading buffer (4x Laemmli buffer or NuPAGE LDS sample buffer with NuPAGE reducing agent (Thermo-Fisher-Scientific)), and were denatured (10 min, 95–100 °C) before separation by SDS PAGE (NuPAGE Novex 4–12% Bis-Tris gels (Thermo-Fisher-Scientific) or 4–20% Mini-PROTEAN® TGX™ precast protein gels (Bio-Rad)) and transfer to 0.2 µm nitrocellulose membranes (Bio-Rad). Membranes were blocked for 1 h in blocking buffer (3% BSA/TBST) and incubated o/n at 4 °C with primary antibody (diluted in blocking solution). Membranes were then washed three times for 15 min with TBST, followed by incubation with secondary antibodies (diluted in blocking solution) and three additional wash steps. Primary antibodies used included: rabbit-anti-MLKL (1:1000; Abcam, ab194699; or Cell Signaling, 14993), rabbit-anti-phospho-S358-MLKL (1:1000; Abcam, ab187091), rabbit-anti-RIPK3 (1:1000; Cell Signaling, 13526), rabbit-anti-Slc1 (1:1000, previously described antiserum [58]), mouse-anti-CASP8 (1:1000; Cell Signaling, 9746), rabbit-anti-CASP3 (1:1000; Cell Signaling, 9665), rabbit-anti-cleaved-CASP3 (1:1000; Cell Signaling, 9661), mouse-anti-β-actin (1:1000; Cell Signaling, 3700), and HRP-conjugated mouse-anti-β-actin (1:50000; Abcam, ab49900). HRP-conjugated secondary antibodies (Southern Biotech or Thermo-Fisher-Scientific) were used at a 1:50000 dilution. Membranes were incubated for 1 min with ECL Prime Western Blotting Detection Reagent (GE Healthcare) or SuperSignal West Pico PLUS Chemiluminescent Substrate (Thermo-Fisher-Scientific) and chemiluminescent signals were recorded with an Image Quant LAS4000 imaging system (Fujifilm). Membranes were stripped with Restore Plus western blot stripping buffer (Thermo-Fisher-Scientific) and blocked before detection of additional targets. Quantification of band intensities was made using Image Studio Lite vs 5.2 (LICOR).

### Determination of infectious progeny

To quantify infectious progeny formation, 96-well plates with confluent HeLa cell monolayers were infected with a low dose of *C. trachomatis* (<50% infected cells) and treated with apoptosis inducers (STS (1 μM), TNF-α (50 ng/ml + 2.5 μg/ml CHX)) at 24 hpi. At various time points post treatment culture supernatants and cell lysates (prepared by H_2_O-based cell lysis [57]) were collected. Infectious particles in the initial inoculum (input) and collected samples (output) were quantified by infecting confluent Vero cell monolayers with serial dilutions, followed by fluorescence microscopic determination of inclusion numbers at 28 hpi (as described previously [57]). From the IFUs detected in the input and the output, the number of infectious particles formed per infected cell could be determined.

### Statistics

Statistical analysis was performed using the software GraphPad Prism 6.01 (*p < 0.05; **p < 0.01; ***p < 0.001; ns, not significant). Samples sizes (n) indicated in figure legends refer to the number of independent experiments (biological replicates).

## Electronic supplementary material

Supplementary material

Movie S1

Movie S2

Movie S3

Movie S4
