# Supplementary material for: Chlamydia trachomatis fails to protect its growth niche against pro-apoptotic insults
Source: Cell Death Differ. 2018 Oct 30;26(8):1485–500. doi: 10.1038/s41418-018-0224-2 (PMC6748135; doi:10.1038/s41418-018-0224-2)
Supplement: Supplementary file 1 — Supplementary material [file 41418_2018_224_MOESM1_ESM.pdf]

Supplementary Figures

Figure S1

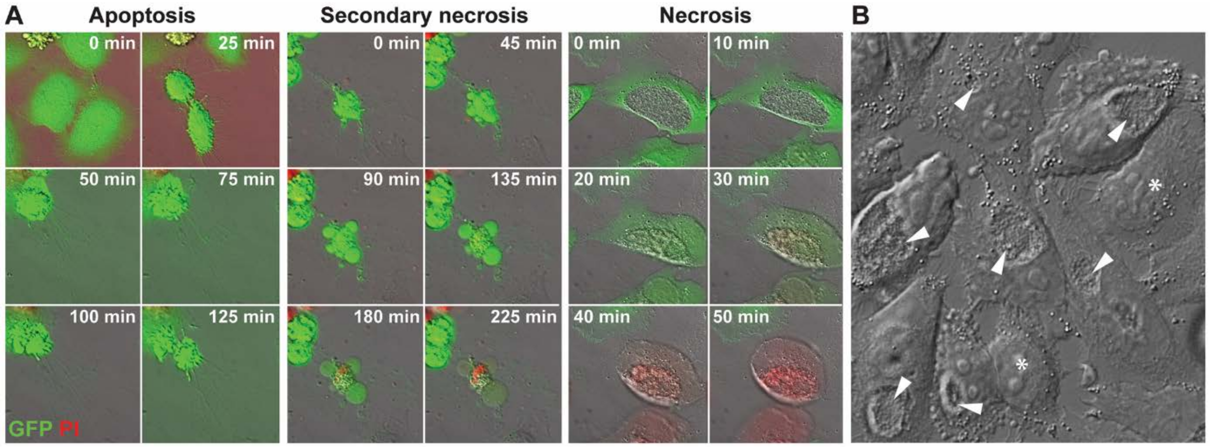

Distinction between apoptotic and necrotic cells and between uninfected and infected cells based on morphological traits in live cell microscopy. (A) Representative image series from time lapse movies illustrating examples of apoptosis (left), secondary necrosis of apoptotic cells (middle), and necrosis in absence of typical apoptotic morphological features (right). Uninfected (left, middle) and infected (5 IFU/cell) GFP-expressing HeLa cells were monitored by time lapse microscopy in a medium containing propidium iodide (PI, 1 µg/ml). Cell death was induced with TNF-α (50 ng/ml + 2.5 µg/ml CHX). The time indicated in each image represents the time elapsed in respect to the first image of the respective image series. Note that plasma membrane rupture, here also detectable as loss of GFP and influx of PI, could also be inferred based solely on changes in cell morphology. **(B)** Representative image from a live cell movie illustrating examples of uninfected (inclusion-free; asterisks) and infected (inclusion-containing; arrowheads) cells in an infected HeLa cell culture (5 IFU/cell, 26 hpi).

Figure S2

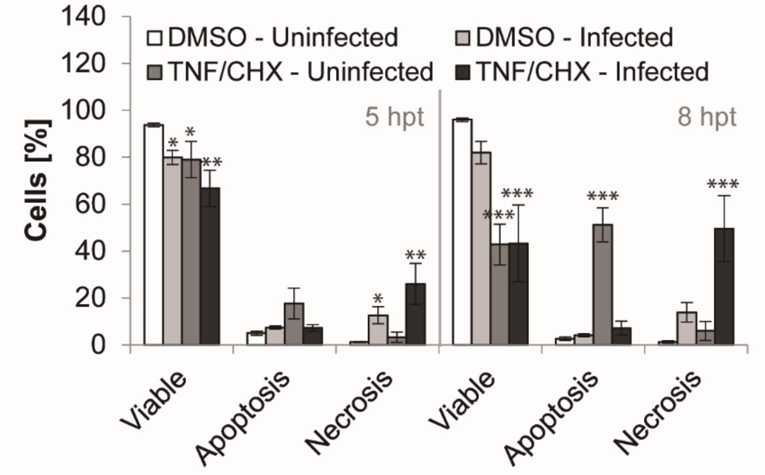

Annexin V/PI staining confirms that infected HeLa cells die from necrosis in response to pro-apoptotic stimuli. Uninfected and infected (10 IFU/cell, 24 hpi) HeLa cells were treated with TNF-α (50 ng/ml + 2.5 µg/ml CHX) or DMSO (mock). At 5 or 8 hpt cells were stained (Annexin V-AlexaFluor488, PI, and Hoechst 33342) and imaged. Fluorescence intensity thresholds were defined to classify each cell as either viable (Annexin V/PI-double negative), apoptotic (Annexin V-positive/PI-negative), or necrotic (PI-positive) (mean±SD, n=3, ANOVA).

Figure S3

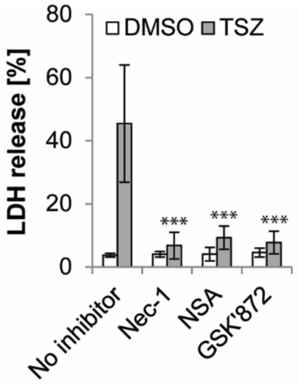

Pharmacologic inhibitors of necroptosis block experimentally induced necroptosis in HT29 cells. Necroptosis was induced in HT29 cells with TSZ (a mixture of TNF-α (20 ng/ml), Smac mimetic BV6 (1 µM), and Z-VAD-FMK (50 µM)). Inhibitors (GSK’872 (8 µM), necrosulfonamide (NSA, 2 µM) and necrostatin-1 (Nec-1, 40 µM)) were added prior to induction. LDH activity in culture supernatants was measured at 9 hpt (mean±SD, n=3, two-way ANOVA).

Figure S4

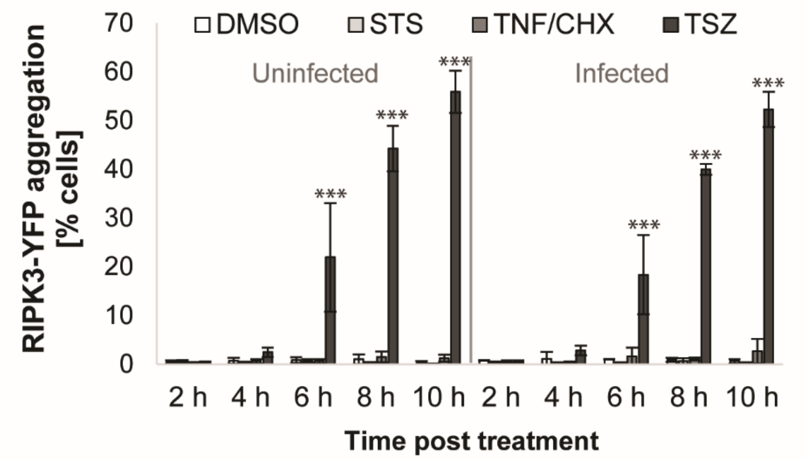

A time course analysis confirms that apoptosis inducers fail to induce RIPK3 activation in *Chlamydia*-infected cells. Infected (10 IFU/cell, 24 h) and uninfected HT29 cells expressing RIPK3-YFP were treated with TSZ (a mixture of TNF-α (20 ng/ml), Smac mimetic BV6 (1 µM), and Z-VAD-FMK (50 µM)), STS (1 µM), or TNF-α (200 ng/ml + 2.5 µg/ml CHX). At indicated time points cells were fixed, stained (Hoechst 33342 and HCS), and imaged on a Cellomics ArrayScan VTI HCS imaging system. The percentage of RIP3K-aggregation-positive cells was determined based on a fluorescence intensity threshold that was inferred from the observed intensity in the controls (untreated and TSZ-treated uninfected cells) (mean±SD, n=3, ANOVA).

Figure S5

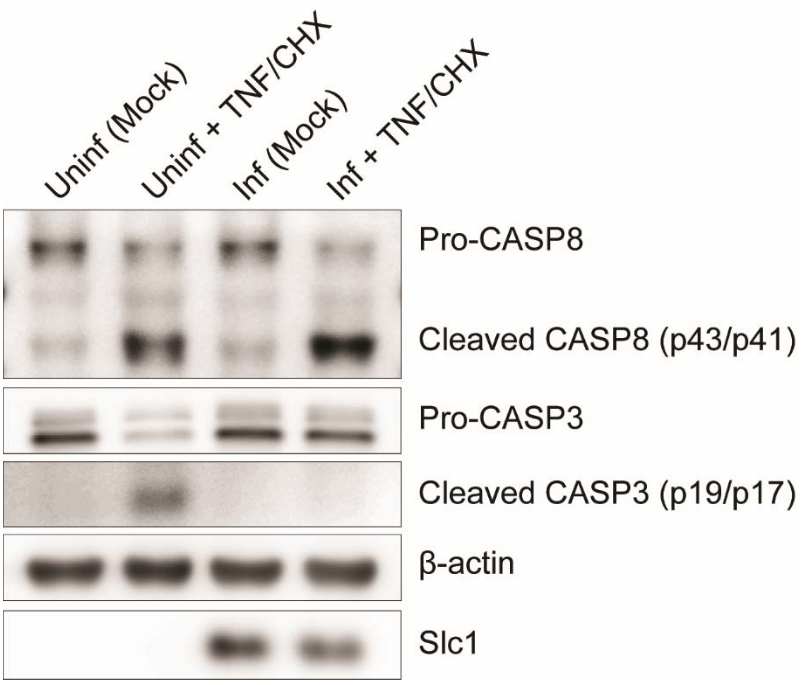

TNF/CHX induces CASP8 activation, but not CASP3 activation, in *Chlamydia*-infected cells. Infected (10 IFU/cell, 24 h) and uninfected HeLa cells were treated with TNF-α (50 ng/ml + 2.5 µg/ml CHX). Protein samples for western blot analysis were generated at 7 hpt. The displayed blot is representative for 3 independent experiments.

Supplementary Movies

Movie S1: Time lapse microscopic monitoring of uninfected and *C. trachomatis*-infected HeLa cells in absence of pro-apoptotic stimulation. Uninfected (left) and *Chlamydia*-infected (5 IFU/cell, right) HeLa cells were treated with DMSO (mock-treated) and monitored by time lapse microscopy until 17 hpt. Asterisks indicate examples of inclusions.

Movie S2: Time lapse microscopy displaying the induction of apoptotic morphologies in uninfected and necrotic cell death in *C. trachomatis*-infected HeLa cells in presence of TNF/CHX. Uninfected (left) and *Chlamydia*-infected (5 IFU/cell, right) HeLa cells were treated with TNF-α (50 ng/ml + 2.5 µg/ml CHX) and monitored by time lapse microscopy until 17 hpt. Asterisks, arrowheads, and arrows indicate examples of inclusions, necrotic cells and apoptotic cells, respectively.

Movie S3: Time lapse microscopy displaying the induction of apoptotic morphologies in uninfected and necrotic cell death in *C. trachomatis*-infected HeLa cells in presence of ActD. Uninfected (left) and *Chlamydia*-infected (5 IFU/cell, right) HeLa cells were treated with ActD (1 µM) and monitored by time lapse microscopy until 17 hpt. Asterisks, arrowheads, and arrows indicate examples of inclusions, necrotic cells and apoptotic cells, respectively.

Movie S4: Time lapse microscopy displaying the induction of morphologies alterations and cell death in uninfected and necrotic cell death in *C. trachomatis*-infected HeLa cells in presence of STS. Uninfected (left) and *Chlamydia*-infected (5 IFU/cell, right) HeLa cells were treated with STS (1 µM) and monitored by time lapse microscopy until 17 hpt. Asterisks, arrowheads, and arrows indicate examples of inclusions, necrotic cells and apoptotic cells, respectively.
